# Promoter hypermethylation of *Wnt* pathway inhibitors in hepatitis C virus - induced multistep hepatocarcinogenesis

**DOI:** 10.1186/1743-422X-11-117

**Published:** 2014-06-20

**Authors:** Muhammad Umer, Sohail Asif Qureshi, Zahid Yasin Hashmi, Asif Raza, Janbaz Ahmad, Moazur Rahman, Mazhar Iqbal

**Affiliations:** 1 Health Biotechnology Division, National Institute for Biotechnology and Genetic Engineering (NIBGE), Faisalabad 38000, Pakistan; 2 Syed Babar Ali School of Science & Engineering, Lahore University of Management Sciences, Sector-U, DHA, Lahore, Pakistan; 3 Department of Medicine, Punjab Medical College, Faisalabad 38800, Pakistan; 4 Liver Center, District Headquarter Hospital, Faisalabad 38000, Pakistan; 5 Department of Pathology, Punjab Medical College, Faisalabad 38800, Pakistan

**Keywords:** Hypermethylation, *Wnt* pathway, HCV, SFRP2, DKK1, Hepatocarcinogenesis

## Abstract

**Background:**

Aberrant DNA methylation profiles are a characteristic feature of almost all types of cancers including hepatocellular carcinoma (HCC) and play an important role in carcinogenesis. In spite of the accumulating evidence that suggests appearance of such aberrations at precancerous stages, very little effort has been invested to investigate such possible methylation events in patients at risk of developing HCC i.e. those suffering from chronic hepatitis C virus (HCV) infection and liver cirrhosis (LC). We reasoned that such an analysis could lead to the identification of novel predictive biomarkers as well as potential drug targets.

**Methods:**

Promoter methylation status of two *Wnt* inhibitors SFRP2 and DKK1 was quantitatively analyzed by bisulfite pyrosequencing in a series of liver biopsy samples. These biopsies were collected from HCV-infected individuals suffering from chronic hepatitis (CH; n = 15), liver cirrhosis (LC; n = 13) and hepatocellular carcinoma (HCC; n = 41). DNA isolated from infection free normal livers (N; n =10) was used as control.

**Results:**

Our analysis revealed that both of the genomic loci were significantly hypermethylated in CH patients’ livers as compared to normal controls (*p* = 0.0136 & 0.0084 for SFRP2 and DKK1, respectively; Mann–Whitney U test). DNA methylation levels for both loci were also significantly higher in all the diseased cohorts as compared to normal controls (*p* < 0.0001 and = 0.0011 for SFRP2 and DKK1, respectively; Kruskal-Wallis test). However, a comparison between three disease cohorts (CH, LC & HCC) revealed no significant difference in levels of DNA methylation at DKK1 promoter. In contrast, a progressive increase in DNA methylation levels was observed at the SFRP2 promoter (i.e. N < CH & LC < HCC).

**Conclusion:**

This study demonstrated that in HCV infected liver tissues hypermethylation at promoter regions of key cancer related genes SFRP2 and DKK1, appears early at CH and LC stages, long before the appearance of HCC.

## Background

Hepatocellular carcinoma (HCC) is the sixth most common cancer and is the third leading cause of cancer related mortality worldwide. According to International Agency for Research on Cancer (IARC) reports 696,000 people died of liver cancer in the year 2008 alone [1]. Multiple factors have been associated with HCC, however hepatitis B & C virus (HBV and HCV) infections are considered to be the major underlying etiology, as together these viruses account for more than two-thirds of HCC cases worldwide [2]. With around 2 billion people infected with HBV worldwide, of which approximately 350 million are chronic HBV carriers [3], and 170 million infected with HCV [4], HCC poses a major health concern globally. Recent estimates show that at least 15 million people in Pakistan are infected either with HBV or HCV (2.5% and 4.8% of the total population, respectively) [5,6], and are hence at an increased risk of developing HCC.

HCV induced HCC follows a progressive course of development from hepatitis to HCC in most cases (i.e., hepatitis → fibrosis → cirrhosis → HCC) [7]. Even though various aspects of this complicated pathogenesis have been interrogated, its underlying mechanism(s) remain elusive. Since HCV is different from HBV, as it harbors an RNA genome that does not integrate in the host cell genome [8], therefore, alternative or indirect models of HCV mediated hepatic oncogenesis have been proposed. It has been suggested that immune mediated chronic liver damage induced by persistent HCV infection, and the accompanying compensatory hepatic regeneration by proliferation and cell division, might culminate in a microenvironment that is conducive for increased mutagenic rates [9]. However, the development of HCC in transgenic mice expressing the HCV core gene alone suggests that alternative mechanisms may also be involved [10]. It has been proposed that HCV induces hepatocarcinogenesis via host and viral protein interactions [11]. A number of studies using mice engineered to carry various HCV genes have demonstrated that their respective expression might promote HCC by several mechanisms including inhibition of apoptosis, pro-oncogenic pathway activation and increased production of reactive oxygen species [11]. While these studies highlight the role of HCV proteins as tumor promoters, it remains an open question that whether or not intracellular expression of these proteins triggers hepatic neoplasm.

Alterations in the normal DNA methylation patterns are found ubiquitously in most types of cancers and play a fundamental role in genesis of cancers including HCC [2,11-13]. For example, activation of canonical *Wnt/ß*-Catenin pathway which is implicated in almost all types of cancers [14-19], has often been attributed to epigenetic silencing of *Wnt* inhibitors like SFRP2 and DKK1 [20-24]. Although whole genome sequencing of HCC tissues has found etiology-specific recurrent mutation patterns as well as key pathways that might be altered as a result of these genetic alterations [14,15], absence of such mutational aberrations in precancerous lesions indicates that they might appear late in this multistep carcinogenesis. In contrast, it has been suggested that epigenetic aberrations such as histone modification and/or DNA methylation might serve as key triggers that initiate carcinogenesis [25]. Some studies have reported the promoter DNA hypermethylation of tumor suppressor genes in pre-cancerous lesions like chronic hepatitis, cirrhosis in liver [26,27] and atypical hyperplasia in breasts [28], highlighting the early onset of epigenetic dysregulations in multistep oncogenic processes.

Aberrant DNA methylation patterns are a promising biomarker for early detection and assessment of future cancer risk in high risk populations owing to their early appearance in carcinogenesis [29]. However, very few studies exist in which methylome alterations in liver tissues obtained from HCV infected non-cancer patients (CH & LC) have been explored [30]. Moreover, in spite of the near ubiquitous *Wnt* pathway activation [14,15] and possible epigenetic silencing of *Wnt* inhibitors, especially SFRP2 and DKK1 in HCC [21-24], very limited data is available regarding methylation of these loci in HCV infected non-cancerous patients. To the best of our knowledge, no studies till date have systematically analyzed promoter methylation levels of these important *Wnt* inhibitors (SFRP2 & DKK1) in HCV infected CH & LC patients (without cancer) and compared these methylation levels with infection free normal liver tissues as well as HCC.

In this study, we tried to fill this gap by carrying out DNA methylation analysis of SFRP2 and DKK1 promoter regions, using a series of biopsies obtained from HCV infected CH and LC patients. These patients did not exhibit any signs of presence of tumorous mass on their livers. DNA obtained from infection free normal liver tissues as well as biopsy samples from an independent group of HCV-positive HCC patients were also included for comparative analysis. Our results demonstrate that both promoters experience elevated levels of DNA methylation in HCV infected CH, LC and HCC patients as compared to normal controls, indicating that promoters hypermethylation of these key cancer related genes (SFRP2 & DKK1) is an early event in precancerous HCV infected liver tissues.

## Results

### Patient characteristics

Among the 15 chronic hepatitis patients included in this study, histopathological analysis revealed minimum or no fibrosis in all collected samples. Out of the 15 suspected liver cirrhosis patients, 13 were finally selected for this study as histopathology reports for 2 patients were not available. Similarly, out of the 50 HCC patients initially biopsied, only 41 were included in final DNA methylation analysis after histopathological analysis confirmed that biopsied tissue corresponded to tumorous growth. Owing to co-morbidities that may arise in advanced HCC patients and the resulting difficulties and contraindications related to biopsy procedure, HCC patient group was largely uniform in its composition with regard to tumor stage. All the biopsied patients had 1–3 solitary tumorous growths that were ≤ 4 cm in size (Stage 0/A, BCLC staging system [31]). α-Fetoprotein levels were highly variable and correlated poorly with stage or severity of disease. Basic demographic and clinicopathological data of patient groups are shown in Tables 1 and 2, respectively.

**Table 1:** Patients demographic data

	**Chronic hepatitis**	**Liver cirrhosis**	**Hepatocellular carcinoma**
	**(n = 15)**	**(n = 13)**	**(n = 41)**
**Age (years)**			
< 40	07	04	03
40-60	08	07	27
> 60	00	02	11
**Gender**			
Male	09	10	32
Female	06	03	09

**Table 2:** HCC patients’ clinicopathological features

**Variable**	**Number of patients**
**Tumor stage (BCLC staging system)**	
Stage 0	17
Stage A	24
**Number of nodules**	
Unilocular	11
Multilocular (<4)	30
**Background cirrhosis**	
Yes	34
No	07
**Alpha-fetoprotein level**	
< 20 ng/ml	06
20-400 ng/ml	23
> 400 ng/ml	12

### Promoter DNA methylation analyses of normal liver samples

**Figure 1:**
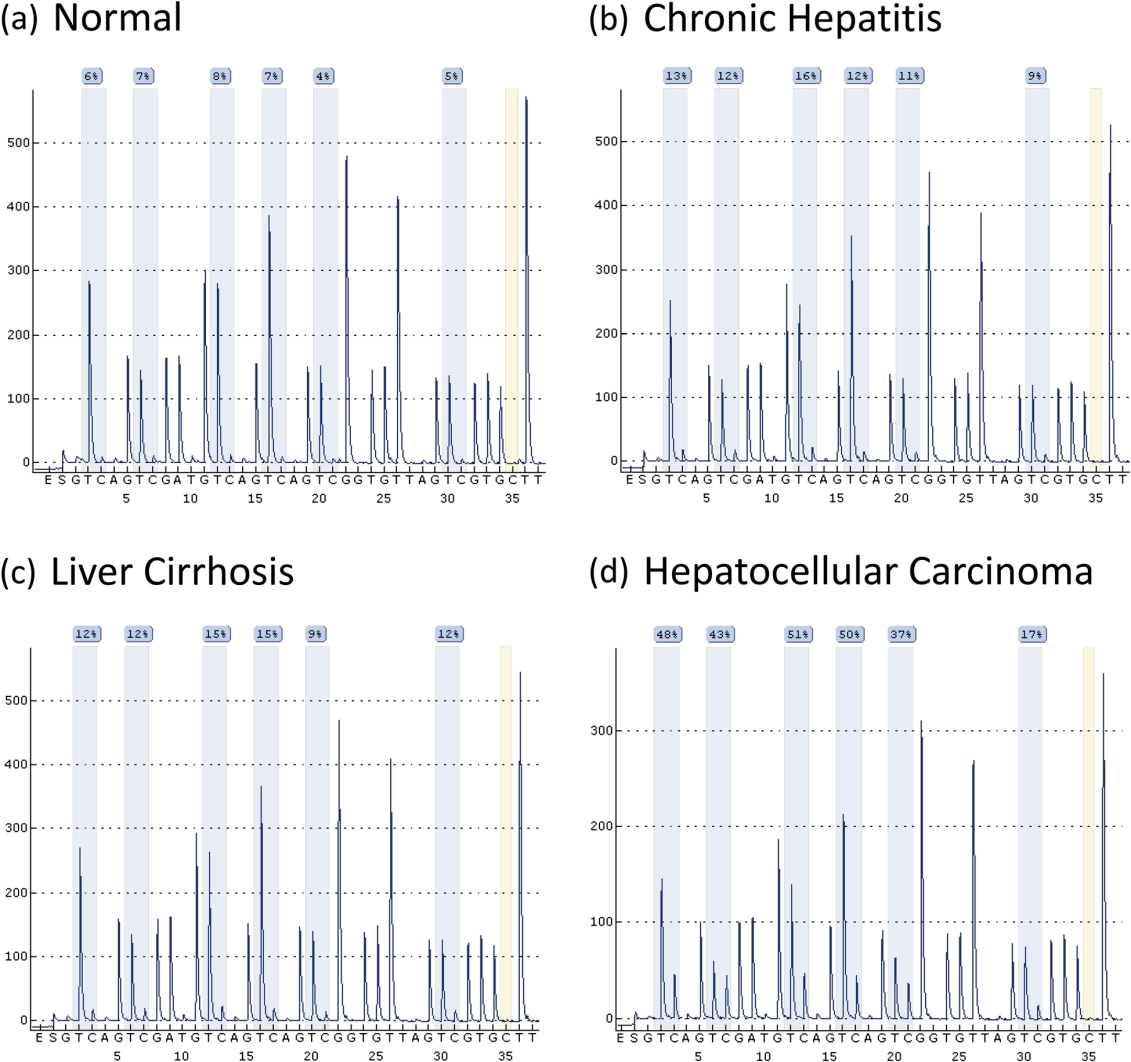
**Representative pyrograms of SFRP2 promoter DNA methylation analysis. (a)** Normal liver samples **(b)** Chronic hepatitis **(c)** Cirrhotic liver tissue **(d)** HCC tissue specimen.

**Figure 2:**
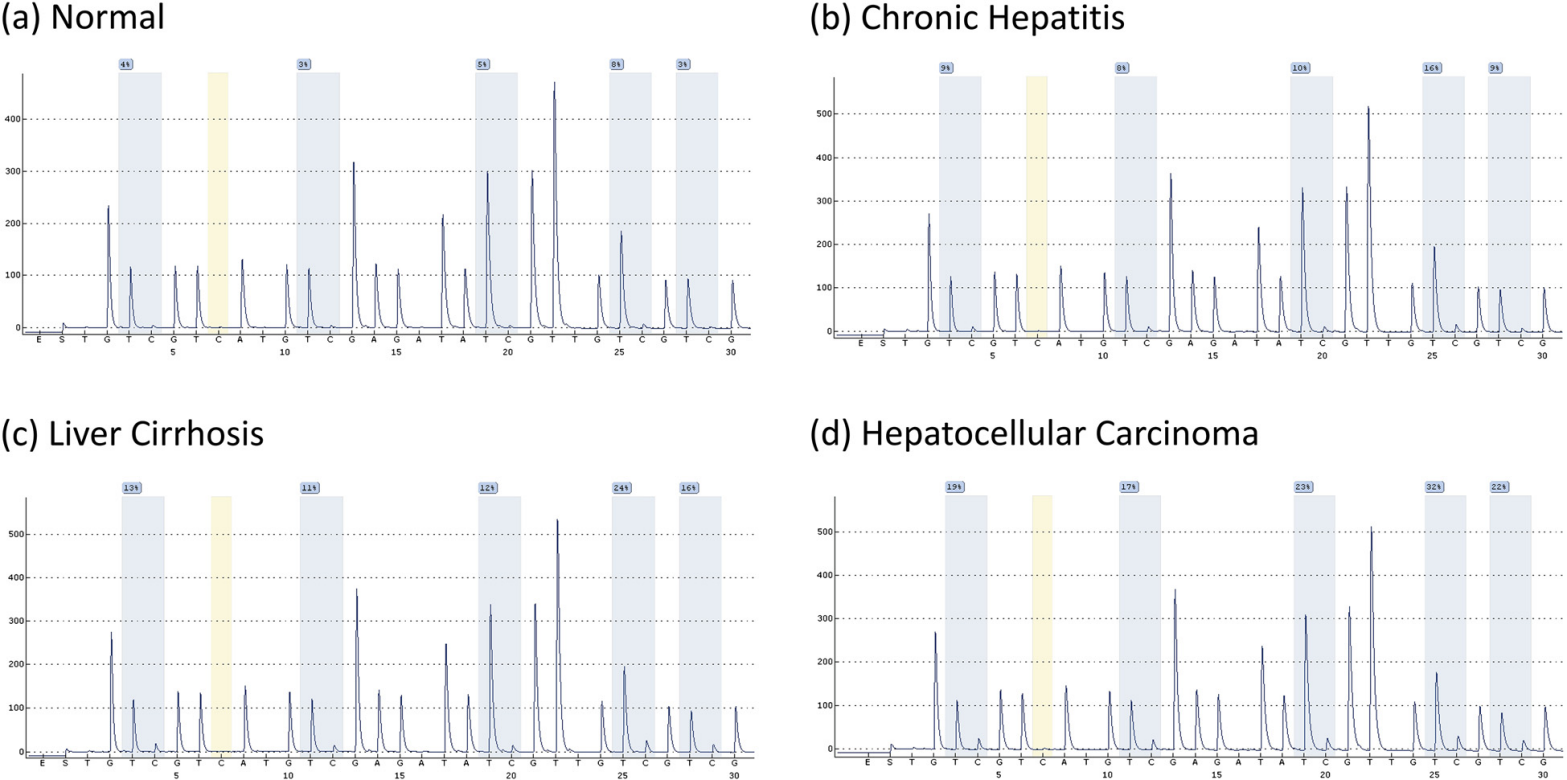
**Representative pyrograms of DKK1 promoter DNA methylation analysis. (a)** Normal liver samples **(b)** Chronic hepatitis **(c)** Cirrhotic liver tissue **(d)** HCC tissue specimen.

**Figure 3:**
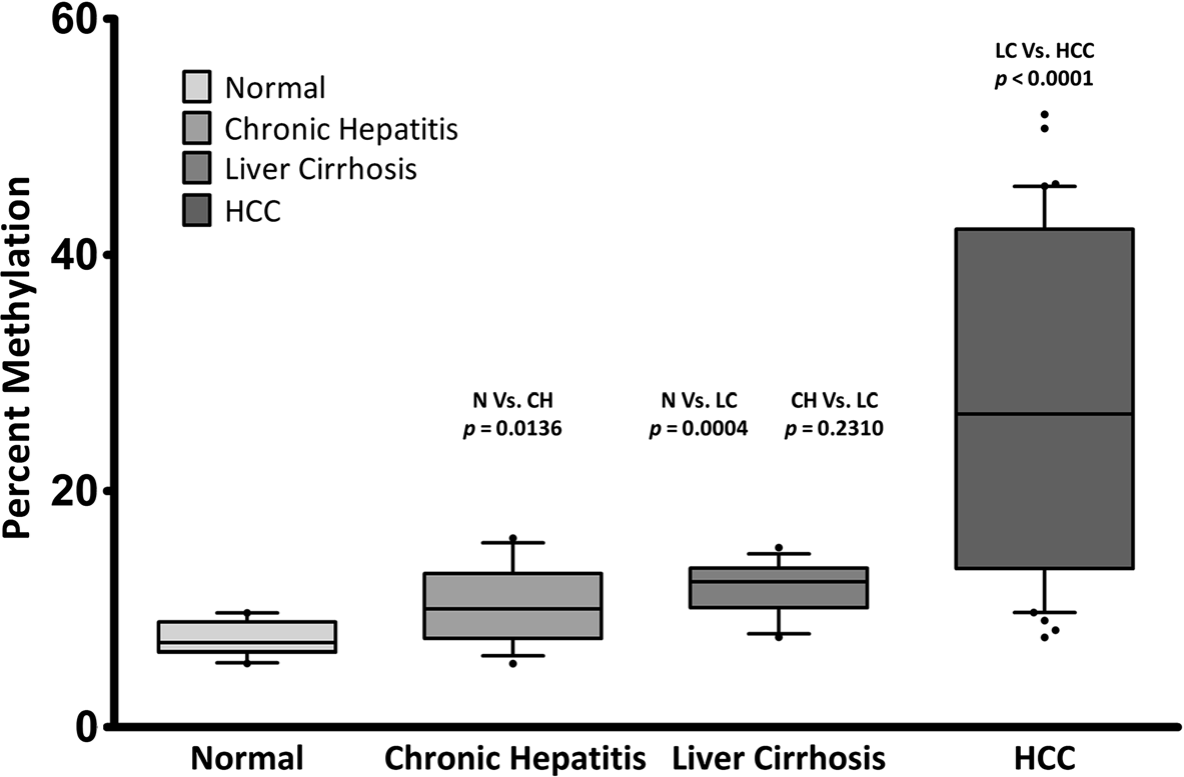
**SFRP2 promoter DNA methylation analysis.** Box and whiskers representation of average DNA methylation percentages at SFRP2 promoter region. Methylation levels increased in a progressive fashion that coincided with the progression of liver disease from chronic hepatitis to HCC. Significance of difference in methylation level calculated by Mann–Whitney test (*p* values) are shown where relevant. For more detailed information about group-wise comparison, see Table 3. N = Normal, CH = Chronic Hepatitis, LC = Liver Cirrhosis, HCC = Hepatocellular Carcinoma.

**Figure 4:**
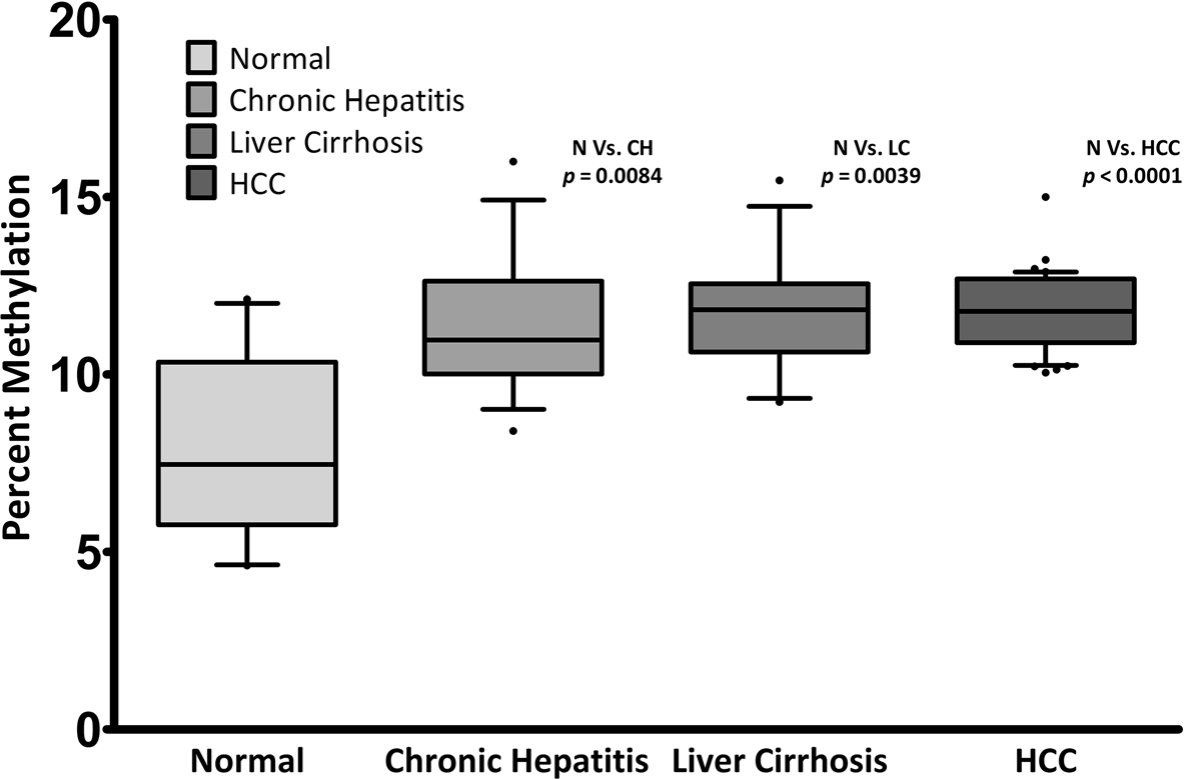
**DKK1 promoter DNA methylation analysis.** Average methylation percentages in diseased cohorts were significantly higher than normal livers (*p* < 0.05, Kruskal-Wallis test). However the three diseased cohorts did not differ from each other significantly. (See Table 3 for more details). N = Normal, CH = Chronic Hepatitis, LC = Liver Cirrhosis, HCC = Hepatocellular Carcinoma.

The pyrosequencing based assays employed in our study were able to quantify methylation at each of the 5 consecutive CpG positions in both of the promoter regions studied. Example pyrograms are shown in Figures 1 and 2. Our overall analysis revealed that both of the target regions generally remained hypomethylated in normal livers. The baseline methylation levels in normal controls for SFRP2 promoter region ranged from 5.4 to 9.7 (mean ± SD, 7.4 ± 1.5; Figure 3). Similarly average percentage methylation across 5 CpG positions investigated for DKK1 promoter sequence ranged from 4.6 to 12.1 (mean ± SD, 8.1 ± 2.6; Figure 4).

### SFRP2 promoter region shows a progressive hypermethylation pattern in multistep hepatocarcinogenesis

SFRP2 promoter methylation level was analyzed in liver biopsy samples obtained from HCV-positive patients suffering from chronic hepatitis (n = 15) or liver cirrhosis (n = 13), without any concomitant tumorous growth on their livers. It was observed that levels of methylation at the interrogated loci were significantly higher in both chronic hepatitis as well as liver cirrhosis groups as compared to normal livers (*p* = 0.01 & 0.0004, respectively; Mann–Whitney test). Average percentage methylation across the 5 consecutive CpG positions analyzed ranged from 5.4 to 16 (mean ± SD, 10.5 ± 3.2) for chronic hepatitis samples while for samples obtained from cirrhotic livers the values ranged between 7.6 and 15.2 (mean ± SD, 11.8 ± 2.2).

While values for all other tissue types varied between a narrow range samples obtained from HCC patients exhibited a high variance in methylation percentage (co-efficient of variation 52.5%). Average percent methylation in HCC samples ranged between 7.6 and 52 (mean ± SD, 27.5 ± 14.5). A comparison of HCC group with other groups (i.e., N, CH & LC) revealed that HCC group shows a significantly higher level of methylation in comparison to all the other groups (HCC Vs LC, *p* = 0.001), *p* values computed by Mann–Whitney test for group wise comparison are shown in Table 3 (also see Figure 4).

**Table 3:** Comparison of patient groups for significance of difference in methylation

**Comparison**	**SFRP2**	**DKK1**	**Significance**	
			**SFRP2**	**DKK1**
**Normal vs Chronic hepatitis**	0.0136	0.0084	Yes	Yes
**Normal vs Liver cirrhosis**	0.0004	0.0039	Yes	Yes
**Normal vs HCC**	<0.0001	<0.0001	Yes	Yes
**Chronic hepatitis vs Liver cirrhosis**	0.2310	0.6784	No	No
**Chronic hepatitis vs HCC**	<0.0001	0.2832	Yes	No
**Liver cirrhosis vs HCC**	0.0013	0.7462	Yes	No

Table 3 summarizes the group wise comparisons carried out using non parametric two tailed Mann–Whitney U test.

### DKK1 promoter hypermethylation is an early event but does not show progressive increase in HCV mediated multistep hepatocarcinogenesis

DKK1 promoter region was also found to be hypermethylated in liver biopsies obtained from HCV infected patients at various stages of disease progression as compared to normal controls (*p* = 0.001; Kruskal-Wallis test). Mann–Whitney test was employed to compare the diseased groups (CH, LC, HCC), which revealed that no significant differences in level of methylation between them are found for DKK1 promoter, in contrast to SFRP2 (see Figure 2 and Table 3).

## Discussion

DNA methylation is considered as an important contributor to the development of HCC. However in spite of the growing body of evidence which suggests that it is an early event in hepatocarcinogenesis [32-35], identification of important methylation events in diseased conditions of liver (CH & LC), which might predispose affected patients to the development of HCC later in life, has not been studied extensively. Recent cell culture studies point towards a possible direct role of HCV core protein in modulating host cell methylome [36,37]. Therefore it will be important to investigate whether patients infected with HCV, yet without HCC, harbor any aberrations in DNA methylation patterns in their livers. Most of the studies aiming to identify early changes in methylation patterns make use of histologically non-malignant liver tissues obtained from HCC patients [32,33,38,39]. However, as viral induced HCC develops after decades of infection, this strategy might not prove to be adequate because it will be difficult to differentiate between “driver” and “passenger” methylation [40] aberrations in such long infected hepatocytes, even if they appear histologically non-malignant. Therefore, in this study we set out to explore possible methylation aberrations in HCV infected non-cancerous patients (CH & LC) with the aim to identify DNA methylation markers which can be exploited for surveillance and carcinogenic risk estimation in high risk populations as well as development of novel drug targets.

Our analysis revealed that both of the *Wnt* inhibitors included in this study display an increased level of methylation in their promoter regions in HCV infected patients even at the very early stages of hepatic disease (Figures 4 & 2, Table 3). Chronic hepatitis tissue samples exhibited significantly higher levels of methylation as compared to normal controls (*p* = 0.0136 & 0.0084 for SFRP2 & DKK1 respectively, Mann–Whitney U test). CH patients included in our study had minimal liver damage and were ideal candidates for interferon therapy. Occurrence of promoter hypermethylation in such early stage liver disease, points to the fact that this epigenetic aberration might occur much earlier than previously thought [33]. Similarly, the other non-cancerous disease cohort i.e. LC also showed significantly higher methylation levels as compared to normal controls (see Table 3 for more details). It is important to note here that although the methylation percentages in CH and LC groups differed significantly from normal controls, the maximum observed percent methylation did not exceed 16% for both the regions studied, in both of the diseased cohorts. This apparently “low” methylation can be attributed to the fact that sample DNA was obtained from a heterogeneous population of infected and non-infected hepatocytes. As previous studies have reported that only a very small percentage of hepatocytes (2-30%) are actually infected with HCV [41,42], the said “low” methylation observed in our experiments appears to be in agreement with findings of these studies.

Interestingly SFRP2 promoter exhibited a pattern of progressive increase in DNA methylation that coincided with the various stages of liver disease, i.e. chronic hepatitis and cirrhotic samples exhibited methylation levels which were statistically higher than normal controls (N Vs CH *p* = 0.0136, N Vs LC *p* = 0.0084, Mann Whitney-U test). Similarly, HCC sample cohort showed significantly higher methylation as compared to chronic hepatitis as well as liver cirrhosis samples (CH Vs HCC *p* < 0.0001, LC Vs HCC *p* = 0.0013). This apparent increasing trend might be because promoter methylation mediated silencing of SFRP2 at the early stages of liver disease provides a definitive selective advantage to affected cells and hence helping in their clonal expansion [30].

One striking observation that emerged from our results was that although DKK1 promoter methylation was found to be significantly higher in all the three diseased groups as compared to normal controls (*p* = 0.001; Kruskal-Wallis test), the three said groups did not differ from each other considerably (See Table 3 for details). Similar results were obtained in an earlier study by Yang *et al.*, [23] which reported that DKK1 promoter methylation in HCC tissues did not differ significantly from surrounding non-tumor tissue or from independent cirrhotic liver samples. However, to the best of our knowledge, it is for the first time that “baseline” methylation in completely normal liver tissues has been reported and compared with that of various disease cohorts. Significance of this comparison is apparent from our own results where we observed a disease dependent hypermethylation as well as those of Yang *et al.* because the said study also found a certain level of hypermethylation in DKK1 promoter in HCC free LC patients (See Figure 3 in the corresponding reference) [23]. Our results speak strongly in favor of use of infection free normal controls in order to put observed methylation percentages in true context.

In a recent study by Nishida et al*.*[30], a subset of early HCC related TSGs (HIC1, GSTP1, SOCS1, RASSF1, CDKN2A, APC, RUNX3, and PRDM2) were also found to be hypermethylated in HCV infected CH patients. However, interestingly SFRP2 promoter methylation was only found in highly progressed HCCs and hence was not analyzed in non-cancerous HCV infected patients. In contrast, our results demonstrate that SFRP2 promoter exhibits hypermethylation in early HCCs as well as HCV infected non-cancerous (CH and LC) samples. These conflicting observations regarding SFRP2 promoter methylation indicate epigenetic as well as genetic heterogeneity that is characteristic of all cancers in general including HCC [43].

Previous *in vitro*[36,37] and *in vivo* studies [44] as well as our own results point out towards a possible direct role of HCV infection and/or intracellular expression of HCV genes in modulating host cell methylome. Although the exact mechanism underlying this possible effect of HCV infection on host epigenome is still poorly understood, evidence has started to emerge which can provide plausible explanations to this hitherto enigmatic phenomenon. An earlier study reported the promoter hypermethylation mediated down regulation of E-cadherin by HCV core protein in cultured hepatoma cells [36]. Quan et al. [37] proposed that HCV core causes epigenetic silencing of *Wnt* inhibitor SFRP1 by modulating the expression and binding of histone deacetylase-1 (HDAC1) and DNA methyltransferase-1 (Dnmt1). In a recent study Okamoto et al. [44] demonstrated that HBV or HCV infected mice with humanized livers exhibit a time dependent genome wide hypermethylation at various gene promoters. The authors claimed that ROS production by innate immune system components like natural killer T-cells contributes to the development of this aberrant methylation profile. Further studies will be needed to explicitly elucidate the possible methylome modulating properties of various HCV proteins. Moreover, comprehensive genome wide analysis of HCC predisposing conditions (CH & LC) is also highly imperative in order to establish a detailed methylation map of such diseased tissues which could be further exploited as biomarker for cancer risk prediction and identification of novel drug targets for prevention of HCC development.

## Conclusions

Our results demonstrate that both promoters of *Wnt* inhibitor genes (SFRP2 & DKK1) experience a significantly elevated levels of DNA methylation in HCV infected non-cancerous disease cohorts (CH & LC) as well as HCC patients as compared to normal controls. These results correlate that promoters hypermethylation of these key cancer related genes (SFRP2 & DKK1) is an early event in precancerous HCV infected liver tissues. While SFRP2 locus becomes increasingly hypermethylated as the disease progresses from CH to HCC, a similar progressive trend was not observed for DKK1 promoter region. Identification of such methylation events in patients at risk of developing HCC later in life might help in development of novel predictive biomarkers as well as cancer prevention strategies. Data presented in this study points towards a possible direct role that HCV infection itself or the intracellular expression of various HCV genes might play in modulating the host cell methylation pattern, however further studies will be needed to clearly elucidate the underlying mechanism.

### Study limitations

In this study, we have not demonstrated the alterations in gene expression which might be correlated with our reported promoter hypermethylation. However, as association of promoter CpG island hypermethylation with silencing of corresponding gene is now an established fact [45] and many studies conducted in the past have demonstrated such a correlation for our target genes i.e. SFRP2 & DKK1 [20,24]. Therefore, we can rightly expect a similar functional implication of DNA hypermethylation, as observed in our study.

Theme of this study was to check the promoter hypermethylation of cancer related target genes (SFRP2 & DKK1) in HCV infected chronic hepatitis, liver cirrhosis and HCC patients and compare that with infection free normal livers. Therefore, exclusion of HBV samples does not affect the overall theme of this study i.e. aberrations in epigenome start to appear in pre-cancerous stages of cancer causing conditions (chronic HCV infection). A separate study, after including the statistically representative number of HCV and HBV infected liver samples of various disease cohorts, may prove useful to further elucidate the underlying mechanism involve in promoter hypermethylation of *Wnt* inhibitors (SFRP2 & DKK1).

## Methods

### Patients

This study was conducted in collaboration with Liver Center, Faisalabad and Punjab Medical College, Faisalabad. The study was approved by Institutional Ethical Review Committee as well as Ethical Committees of collaborating centers. Infection status of patients initially recruited was confirmed by quantitative HBsAg ELISA (Alpha Diagnostic, Texas, USA) and in house qualitative reverse transcription PCR for HCV RNA as described previously [46]. Only HCV positive patients were included for further study. Patients having HBV infection as well as with HBV & HCV co-infections were excluded. Ultrasonographic examination along with liver function tests was used to initially assess the extent and stage of liver disease. Final grouping of patients in various diseased cohorts was done based on histopathological analysis (see below). As an additional diagnostic parameter, α-Fetoprotein levels in the serum of HCC patients were also determined. Diabetic patients (HbA1c level ≥ 1% above the normal range) and patients with fatty liver disease or marked ascites were also excluded. All patients were tested for possible impaired hemostasis and platelet count. Individuals with PT-INR above 1.5 and platelet count less than 150,000/μl were also not subjected to percutaneous biopsy procedure [47]. A total of 80 liver biopsies were obtained (CH = 15, LC = 15, HCC = 50) between the years 2009 and 2011 from the patients visiting and/or admitted at the collaborating center. Genomic DNA obtained from subjects who had died in accidents and had otherwise histologically normal livers and were free from any of the hepatitis virus infections was used as normal control. These DNA samples were a kind gift from Professor Magnus Ingelman-Sundberg, Section of Pharmacogenetics, Department of Physiology and Pharmacology, Karolinska Institutet, Stockholm, Sweden.

### Collection of liver biopsy samples

After obtaining written informed consent, percutaneous liver biopsies were collected using 18-20G semi-automatic Trucut type biopsy needles (STERICUT® TSK, Japan) under real-time ultrasound guidance by a specialized ultrasonologist according to AASLD guidelines [47]. The biopsy tissue was immediately transferred to PBS solution in sterile containers. The containers were sealed, placed on ice and then quickly transferred to the lab.

### Histopathological analysis of liver biopsies and grouping of diseased cohorts

Patients were divided into three diseased cohorts; Chronic Hepatitis (CH), Liver Cirrhosis (LC) & Hepatocellular Carcinoma (HCC) based on histopathological analysis of liver biopsies. Biopsies were graded/scored using various different scoring systems, depending on the origin of sample. Chronic hepatitis samples were scored using modified HAI [48] while for biopsies showing fibrosis, METAVIR scoring system was used [49]. Only those fibrosis samples were finally included in DNA methylation analyses which showed METAVIR fibrosis score 3–4. HCC tissues were graded based on the degree of tumor differentiation (poorly, moderately & well differentiated).

### Extraction of genomic DNA from liver biopsy samples

Under sterile conditions a small piece from liver biopsy tissue was cut using a sterile surgical blade. The cut piece measured less than ¼th of the total length of biopsy tissue. DNA was isolated using PureLink® Genomic DNA Mini Kit (Invitrogen™) as per manufacturer’s instructions. Larger tissues were fine minced into smaller pieces using sterile scalpel blades. Lysate was prepared by incubating samples at 55°C for 4–10 hours (depending on the size of tissue) in 180 μl PureLink™ Genomic Digestion Buffer (Invitrogen™) in the presence of 20 μl Proteinase-K solution (supplied with the kit). An additional 20 μl Proteinase-K solution (20 mg/ml) was added to the mix after 4 hours of incubation. DNA was eluted in 50 μl elution buffer supplied with the kit.

### Bisulfite conversion and PCR amplification of target regions

Genomic DNA isolated from liver biopsy tissue or that obtained from normal livers (250-500 ng) was used in a 20 μl bisulfite conversion reaction using EpiTect® Bisulfite Kit (Qiagen, Valencia, CA) as per manufacturer’s instructions. DNA was eluted in 20 μl buffer EB and stored at −20°C until further use. Bisulfite converted DNA (1–2 μl) was used in PCR amplification of target regions, which was carried out using PyroMark PCR Kit (Qiagen, Valencia, CA) in a 25 μl reaction volume. A 182 bp region corresponding to SFRP2 promoter region was amplified using primers (Biomers GmbH, Ulm, Germany) reported earlier [50]. SFRP2 reaction mix also included 5 μl Q Solution (supplied with kit). A 161 bp region corresponding to DKK1 promoter region was amplified using Hs_DKK1_01_PM PyroMark CPG assay (Qiagen, Valencia, CA). Reverse primer in both of the cases was biotinylated. Typically ~5 μl of PCR product were analyzed by electrophoresis using a 2% agarose gel prepared in 1X TAE.

### Bisulfite pyrosequencing

Biotinylated PCR products were immobilized on streptavidin-coated sepharose beads (GE Healthcare). The immobilized PCR product was rendered single-stranded on PyroMark Q24 Vacuum Workstation according to manufacturer’s instructions and finally annealed to sequencing primer (final concentration 0.3 μM) by heating at 80°C for 2 min followed by incubation for at least 5 minutes at room temperature [51]. Pyrosequencing reaction was carried out on PyroMark Q24 System. The assay design and analysis of results was carried out using PyroMark Q24 software (version 2.0.6).

### Statistical analysis

Average DNA methylation data (as percent) was analyzed with GraphPad Prism V5.0 (GraphPad Software, San Diego, CA). Significance of difference in percent methylation between diseased cohorts and normal was calculated by nonparametric one way analysis of variance (Kruskal-Wallis test). Group-wise comparisons of percent average methylation between different diseased groups as well as between diseased groups and normal liver tissues were carried out by Mann–Whitney U test.

## Abbreviations

HCV: Hepatitis C virus; HBV: Hepatitis B virus; CH: Chronic hepatitis; LC: Liver cirrhosis; HCC: Hepatocellular carcinoma; ELISA: Enzyme linked immunosorbent assay; RT-PCR: Reverse transcriptase polymerase chain reaction; cDNA: Complimentary DNA; PCR: Polymerase chain reaction; CpG: Cytosine phosphate guanine; HDAC1: Histone deacetylase-1; WHO: World Health Organization; IARC: International Agency for Research on Cancer; OR: Odd ratio; CI: Confidence interval.

## Competing interests

The authors declare that they have no competing interests.

## Authors’ contributions

MU, MI and SAQ conceived and planned the study. Sampling and experimental work were conducted mainly by MU. AR performed USG guided percutaneous liver biopsies while ZYH was involved in clinical examination of patients. JA carried out histopathological analysis of liver biopsy samples. MR shared the cost of research and helped in design of study. MU and MI contributed in manuscript writing while the study was mainly supervised by MI. All authors read and approved the final manuscript.

## Authors’ information

^1^MU PhD student, MR Senior Scientist & MI Principal Scientist/Group Leader at Drug Discovery and Structural Biology, Health Biotechnology Division, National Institute for Biotechnology and Genetic Engineering (NIBGE), Faisalabad - 38000, Pakistan.

^2^SAQ is a Professor and Dean at Syed Babar Ali School of Science and Engineering, Lahore University of Management Sciences, Lahore - 54792, Pakistan.

^3^ZYH is Head at Department of Medicine and Principal Punjab Medical College, Faisalabad - 38800, Pakistan, as well as ^4^Chairman Liver Center, District Headquarters Hospital, Faisalabad - 38000, Pakistan.

^4^AR is working at Liver Center, District Headquarters Hospital, Faisalabad - 38000, Pakistan.

^5^JA during the time period of sampling was working as Head at Department of Pathology, Punjab Medical College, Faisalabad - 38800, Pakistan.
